# Facing yourself in a virtual reality-assisted perspective-taking intervention: an interpretative phenomenological analysis

**DOI:** 10.3389/fpsyg.2025.1547064

**Published:** 2025-06-09

**Authors:** Essi Sairanen, Daniel Wallsten

**Affiliations:** Department of Social and Psychological Studies, Karlstad University, Karlstad, Sweden

**Keywords:** virtual reality, perspective-taking, relational frame theory, interpretative phenomenological analysis, sense of self

## Abstract

**Introduction:**

People's self-narratives—how they relate to themselves and others—are closely intertwined with psychological suffering and wellbeing. This study investigates a perspective-taking intervention rooted in Relational Frame Theory (RFT), where participants observe themselves from an outside perspective in Virtual Reality (VR).

**Methods:**

Three participants watched 3D-filmed versions of themselves through a VR headset while reflecting on perspective-taking questions. Semi-structured interviews were conducted, and the transcripts were analyzed using Interpretative Phenomenological Analysis (IPA).

**Results:**

The intervention helped participants become more aware of their self-relating. They recognized how they internalized perceived judgments from others and how they adjusted their behavior accordingly. Self-critical tendencies and the double standards they applied to themselves vs. others became more apparent, often evoking self-compassion. The intervention also revealed discrepancies between participants' internal and external views—that is, how they perceived themselves from the inside vs. the outside.

**Discussion:**

These findings highlight the promising therapeutic potential of this intervention, both for assessing and influencing self-relating.

## 1 Introduction

The sense of self plays a fundamental role in psychological suffering, with self-criticism and negative self-judgments being central to many psychological problems (Glass et al., 1982; Swallow and Kuiper, 1988). As such, different aspects of “the self” often becomes a key focus in psychological treatments, where clients gain awareness of their own self-relating which potentially allows old self-relating to acquire new meaning (Barnes-Holmes et al., 2020; Hayes, 2004; Kohlenberg et al., 1993). However, influencing self-relating—such as “I am worthless”—can be particularly challenging, as these patterns are often deeply rooted in an individual's learning history and therefore resistant to change.

Relational Frame Theory (RFT; Barnes-Holmes et al., 2001) provides a functional-contextual framework for understanding the verbal processes that constitute a sense of self. This framework offers a basis for predicting and influencing the self through contextual variables. According to RFT, the core element of human cognition is Arbitrarily Applicable Relational Responding (AARRing). RFT posits that verbally able humans relate stimuli in an arbitrary manner—based on socially constructed rules, rather than on formal properties—with the psychological functions of those stimuli being altered accordingly (Hayes et al., 2001). A verbally constructed sense of self involves responding from a me-here-now perspective, which includes distinguishing between me and others, here and there, and now and then (Barnes-Holmes, 2001). The core postulate is that children, as they become verbal, begin to derive their sense of self by distinguishing themselves from others across place and time (Barnes-Holmes et al., 2002; McHugh et al., 2004). Once this perspective is established, it becomes an ongoing behavioral event, participating in virtually every psychological act. In other words, individuals respond to stimuli or events from the constant perspective of a verbal self (i.e., me-here-now or deictic-I). As humans navigate their psychological worlds, the “I” or “me” is related to various qualities, such as male or female, good or bad, valuable or worthless. These qualities can also be related with comparison, such as better or worse, bigger or smaller and so on. Over time, complex relational networks about the self are derived.

Perspective-taking interventions based on Relational Frame Theory (RFT)—such as responding from a me-here-now perspective as if one were you-there-then—can be utilized to influence self-relating (Foody et al., 2013). For instance, observing oneself from the perspective of a loving friend can highlight discrepancies between the standards one applies to oneself vs. others, potentially fostering more compassionate self-relating. Virtual Reality (VR) introduces new possibilities for perspective-taking interventions, and innovative applications are emerging. In a previous study, a perspective-taking intervention grounded in RFT was combined with VR. During the intervention, participants received perspective-taking cues while observing immersive 3D recordings of themselves, allowing them to perceive themselves from an external perspective (Sairanen et al., 2023). The intervention facilitated more positive self-relating; participants tended to relate to themselves more positively immediately after the intervention. However, observations of individual cases revealed variability, with some individuals responding less favorably. Another study explored the use of 3D recordings in VR to influence self-compassion by allowing participants to encounter themselves from an external perspective (Landau et al., 2022; preprint). While the intervention was perceived as meaningful and improved active positive affect, it also led to unintended effects, including reduced self-esteem, diminished self-kindness, and increased self-isolation—particularly among younger participants. These findings underscore the importance of gaining a better understanding of the processes of change involved when using immersive 3D recordings to facilitate new perspectives on the self. They also highlight the need for carefully designed interventions that allow for idiographic exploration in the development of novel therapeutic approaches.

This study aims to explore participants' experiences of a RFT-based perspective-taking intervention that uses immersive VR technology to examine self-relating from an external perspective. Semi-structured interviews are conducted as an integral part of the intervention (e.g., “What was your first impression of the person in front of you?”). An idiographic qualitative approach, Interpretative Phenomenological Analysis (IPA), was chosen to analyze the data, as it facilitates an in-depth examination of individual experiences within their unique contexts (Pietkiewicz and Smith, 2014). Through this interpretative analytical process, researchers aim to understand participants' “lived” experiences by interpreting how they make sense of their world (Pietkiewicz and Smith, 2014; Smith, 2004). IPA is particularly well-suited for exploring dynamic, contextual, and subjective topics, especially those related to identity, the self, and sense-making (Smith, 2004).

By examining how participants relate to themselves during the intervention, this research seeks to provide insight into potential processes of change and to refine perspective-taking interventions assisted by immersive 3D recordings. Ultimately, such interventions may contribute to promoting psychological wellbeing and preventing or treating psychological suffering.

## 2 Methods

### 2.1 Participants

Participants were required to be ≥18 years old. Exclusion criteria included having a psychiatric diagnosis that impaired everyday functioning, uncorrected vision limitations (e.g., not corrected by contact lenses), or insufficient fluency in Swedish.

The final sample consisted of three participants: two females and one male, referred to in this study as Ann, Klara, and Tim. Their ages ranged from 34 to 41 years. All participants were employed and had at least one child living at home.

### 2.2 Materials

#### 2.2.1 The VR intervention

The intervention protocol was based on Sairanen et al. (2023). While observing the 3D-filmed version of themselves, the participants were prompted to respond to me-there-now from me-here-now. Table 1 presents the perspectives and questions that were asked while the participants observed themselves in the 3D virtual space (with clarifying follow-up questions depending on the participant's answers). Each perspective/question was designed to assess how the participants related to themselves, and to themselves with respect to others.

**Table 1:** Perspective-taking prompts in the VR-intervention.

1. [ME-ME; THERE-THEN] What was your first impression of the person in front of you?
2. [ME-ME; THERE-THEN] Can we go back in time to a time and place when the person in front of you could be him/herself and be accepted just as he/she was? When the person could have told or shown just about anything about themselves and still felt fully accepted? Can you tell me about such a time or place?
3. [ME-ME; THERE-THEN] To what extent has the person in front of you hidden or held back parts of themselves throughout life?
4. [ME-ME; THERE-NOW] What do you think the person in front of you is experiencing right now?
5. How do you think the person in front of you perceives themselves right now?
6. [ME-OTHERS; THERE-NOW] If your *mother* was here with us right now. How do you think she would perceive the person in front of you? (repeated with closest friend, father, siblings and partner)
7. [ME-OTHERS; THERE-NOW] If a person who never met the person in front of you was here right now. How do you think he would perceive the person in front of you?
8. [ME-ME; HERE-NOW] How do YOU perceive this person in front of you right now?
9. [ME-ME; HERE-NOW] What are you experiencing right now? (*This question was asked after asking to remove the VR-headset, but keep eyes closed*).

### 2.3 Procedure

The current study was approved by the Ethical Review board at Uppsala University in Sweden [Dnr 2019-06213]. Participants were recruited through Karlstad University's web page and through advertisement in social media. A total of 23 individuals indicated their interest by signing an electronic consent form and filling out a screening survey. The first three individuals who met the inclusion criteria and were reached by the research group were invited to participate in the study.

The VR-intervention included two similar about 60 min sessions that were delivered on two separate occasions during two consecutive days. Initially during both sessions, the participant was filmed with a 3D-camera for 3 min. The participant was advised to remain standing on a spot and keep some natural eye contact with the camera lens. The VR-headset was then applied, the participant got to sit on a chair and watch the filmed version of themselves looped for 20–30 min while they were being interviewed. The same semi-structured interview (see Table 1) were conducted during both VR-sessions with the exception of item 6 in which different people were presented during sessions 1 and 2. Peaceful and calming piano music was played during recording and playback to block out background but also to enhance the emotional aspects of the VR-experience (Barrett et al., 2017; Kaelen et al., 2018; Koelsch, 2014).

### 2.4 Interpretative phenomenological analysis (IPA)

The interview data collected during the VR-intervention sessions was analyzed with IPA. The analysis followed the four-stage process (Pietkiewicz and Smith, 2014; Smith and Nizza, 2022). Analysis began with a close interpretative reading of the first case. The researcher made notes about their observations and reflections. These initial notes were then translated into experiential statements grounded in the particular detail of the participant's account. The next stage involved looking for connections between experiential statements, grouping them together according to conceptual similarities, and providing each cluster with a descriptive label. This resulted in a table/list of major themes and subthemes, and relevant short extracts from the transcript supporting the theme. This process was repeated for each case. After analysis has been conducted on each case, patterns were established cross-case and documented in a master table of themes for the group. The master table was then transformed into a narrative account; the analytic account is supported by verbatim extracts from each participant. The first author conducted the IPA, which was then closely discussed with another author and with students using the data for their master's theses.

## 3 Results

All participants placed significant emphasis on how they were perceived by others. Ann and Klara specifically reflected on their self-esteem, while Tim focused more on social status and fitting into specific social categories. Seven group experiential themes emerged from the analysis of the transcribed interviews, as outlined in Table 2. Despite undergoing the same VR intervention, each participant contributed with both overlapping and unique sense-making around sense of self (i.e., relational responding around self) during the intervention.

**Table 2:** Group experiential themes.

**Group experiential themes**
The burden of thinking about how others perceive me
Adjusting oneself according to social expectations vs. being true to oneself
A mismatch between experiencing oneself from outside and inside
Keeping emotions at bay
A judgmental approach to my own appearance
Increased self-knowledge and acceptance with age
Acknowledging self-criticism fostered the development of self-compassion

### 3.1 The burden of thinking about how others perceive me

All participants exhibited a strong inclination to contemplate their perception and acceptance by others. They reported investing significant time and energy in thinking about how they were perceived by others. This reflection was consistently perceived as burdensome and energy-draining. Notably, Klara provided an explicit account of this phenomenon in the following extract:

She is still very fragile, eh, she cares a lot about what other people think, eh, she's looking for, what can I say, she's looking for some kind of… [sigh] confirmation, ah that, to be enough, like.

Klara perceived herself as fragile in relation to other people. She viewed others as a source of validation for her own sense of worthiness. Similarly, Ann paid a lot of attention to how others saw her and their opinions of her:

I perceive a person who is very self-conscious and who, eh, who kind of thinks a lot, all the time about how she behaves or looks and things like that eh, and that it takes up a lot, that it kind of takes a bit of it, to be present.

This extract highlights the constant presence of other people's perspectives on the self and how it leads to a continuous sense of evaluation. Ann contrasted this ongoing evaluation with the ability to fully be present in the moment, as it diverts attention from other aspects of her experience.

Adolescence emerged as a crucial phase during which individuals experienced a heightened awareness of others' perspectives. This awareness often posed a threat or detriment to their self-esteem and overall sense of identity. The participants' responses varied as adults, with Ann and Klara expressing a lasting impact that was never fully resolved, leaving a sense of permanent “damage”. See the extract from Ann:

An awareness, perhaps, of how one looked and how one was and that it was more important to fit in and then… uh, maybe I didn't come back to this complete security then after that.

In contrast, Tim emphasized the importance of self-development and highlighted the contrast between his current self and his teenage years:

I have a brother with psychiatric diagnosis and I have, when I was younger I thought it was hard and difficult to explain it, that diagnosis and that disease, eh, especially when you were perhaps in, ah but the teenage years like that, then could you sort of find strategies to avoid talking about it.

Participants demonstrated a heightened concern regarding how they were perceived by unfamiliar individuals, viewing it as a means of self-protection. Criticism from others was strongly experienced as aversive and even threatening to their sense of self. In addition, that being connected to the perception of oneself as being “smaller” or “weaker” in relation to others:

I'm becoming quite aware of, in a new social relationship, that I become very like, …what should I say now, what am I thinking, eh that in some way I see myself a little from the outside maybe then. Eh, and that I'm holding back like the spontaneous in some way. (Ann)

She is very concerned about what others think. One, a look from some unknown person who might just happen to be looking can make her think that now there is something wrong with me or, she thinks I have a very strange hat like, uh, so that you are incredible, what should you say, uh, uh, you are in, what should you say preparedness or something that there may be criticism or there may be something that I am not prepared for and, it must not go in like, I think. (Klara)

### 3.2 Adjusting oneself according to social expectations vs. being true to oneself

Participants engaged in reflections on social expectations and norms, noting the various ways in which they adjusted their behavior to fit in. From an external perspective, participants were recognizing and reflecting upon the different strategies they employed to present a particular image to others. Participants expressed a desire to be perceived as self-confident and socially appropriate. Let us look at an extract from Klara:

Here I think she's thinking… here she knows she's being filmed, uh. I thought it was very interesting to see the very first seconds of, because I see this film is looped the very first seconds. Then she stands no, she doesn't make an effort, she's more relaxed on the face, eh. Then she tenses, she pulls the lower jaw forward because she knows she has an overbite (short laugh). So she tenses the lower jaw which also makes the mouth very tight and then so she has a different look than she had at the beginning, eh, as she thinks she, thinks she looks, that she simply looks better, that she should show, as it were, that she is, I am, I have self-confidence and Ah so.

Klara discerned a specific instance in which she became conscious of the external viewpoint and instinctively modified her behavior to project greater confidence. This duality of perspectives involved scrutinizing facial expressions from an external viewpoint while simultaneously connecting them to her internal experience of low self-esteem.

Tim engaged in self-reflection by positioning himself within various social categories. Rather than directly comparing himself to others, he found significance in his connection to specific groups or categories and how well he assimilated within them. By meeting the expectations set by these categories, he experienced a sense of contentment with himself:

But there are some societal categories or some sort of categorization system that you agree on, if you are in this context like; middle class and, uh, ah but parent of small children, and based on those categories you can more or less relate to others and reflect how well one comprehends, fits into the, norm or that type of, category.

Simultaneously, Tim acknowledged the distinction between his personal preferences and the need to conform to social expectations. He made a conscious choice to adjust himself in order to demonstrate to others his affiliation with a particular context or social group:

Here's a person who has made himself, a bit, for their surroundings, uh, like maybe the first choice isn't to, button up the shirt and tuck it into the pants, but it's something that one want to, convey to, high-ranking individuals in another organization that here I belong, sort of in that context, um, so on the surface, it's probably someone who adapts a bit more than he feel is, maybe okay.

Similarly, Klara distinguished between being her authentic self at home and the necessity to adapt and conform in her work environment:

I don't know if I can say that I'm always one hundred percent myself if you're out and about in a work context or if you're, uh, in other types of meetings, eh… but one, ah, I still think that I'm, not quite the same person when I'm at home in my own home, uh, compared to when I go, go outside that door. When I'm at home, but then I'm one hundred percent myself with the flaws that could include….it's sure always like that, like… eh (short laugh) you go and hold your stomach in (short laugh) at work, maybe you don't do it at home, eh, if you feel like you've, ah, put on a little extra weight like, eh, or that you, well, maybe at home you might sit and bite your nails or, (short laugh) scratch your ear or nose or something, but you don't do that in a work context.

Participants reported experiencing a sense of liberation from external expectations and a genuine expression of themselves in their relationships with partners or best friends. Klara found solace in being able to be her authentic self with her partner, as he possessed a deep understanding of her:

I still feel that I can be, myself with, no need to pretend or I can be, ah, he knows my weaknesses and he knows mine, ah, he knows me inside and out.

Similarly, Tim described a friendship where there were no barriers or expectations, allowing him to be completely himself without constraints:

There's like nothing one need to… don't need to uh, reflect so much before like conversations or weigh your words, but, there's like nothing, uh, what can I say, no barriers so, uh, I kind of imagine that it's a feeling like, trust so uh, I was going to say nakedness but that's the wrong word because it's like not that you're vulnerable, but like more that, there's like no demands in this meeting.

A distinct difference emerged between Tim and Klara in their self-perception. Klara described holding a concept of a “true self” and, in certain contexts, experienced a contrast between the image she presented to others and her authentic self. This led her to feel as though she was pretending to be confident when she did not genuinely feel that way. In contrast, Tim viewed himself as being in constant interaction with his environment, acknowledging that his experiences were shaped by external circumstances. He recognized that how others perceived him was contingent on the context, such as through comparisons with others who were present.

### 3.3 A mismatch between experiencing oneself from outside and inside

Participants consistently conveyed a sense of discrepancy between how they were perceived from an external standpoint and their internal experiences. This incoherence was evident in the contrast between their perception of how others viewed them and their own subjective understanding of themselves. Additionally, this mismatch was also observed when participants saw themselves from an external perspective in the VR-environment.

Both Ann and Klara articulated a sense of incoherence between how they were perceived by others and their internal experience of self-esteem or confidence. The perception of oneself as either strong or vulnerable/insecure fluctuated depending on the perspective Ann adopted or the specific aspects of herself she related to. Similarly, Klara described a discrepancy between her outward appearance of calmness and confidence and her inner reality of fragility:

I think sure that, she, she looks, she looks pretty confident, sure of her thing, uh and uh, yes, but rather convincing, um, maybe a little, ah, but, also a little angry, on the verge of, maybe cold uh, expression, um, and I think she feels… um I, I think she feels confident but it's hard to answer that because I know she's fragile inside.

Klara and Ann exhibited distinct responses to how they were perceived as “kind” by others. Klara expressed surprise and worry when she observed herself appearing angry and judgmental, driven by her fear of being judged by others. For Klara, being seen as kind possibly served as a means to counteract this perception and seek acceptance and likeability in social settings:

I'm a little, worried that if it's someone who is maybe, who has, ah but little like myself, that looks and other things eh that people interpret it eh that, that I can do, that I can make someone afraid.

Conversely, Ann was taken aback by her own perceived kindness. For Ann, “kindness” may have been viewed as contrasting with qualities such as strength and being interesting, which she considered more significant in social interactions:

My first impression is that, uh (laughter) it's a person (laughter) who looks surprisingly kind. It probably has to do with the self-image then that I don't think that (laughter) I look so kind and I think the person on the picture still does.

During the VR intervention, participants began to develop a heightened awareness of the inconsistencies within their self-image and started to construct a more nuanced understanding of themselves. As the intervention progressed, Ann, for instance, started to question her own perception of herself and how it differed from the feedback she received from others. This included contemplating the disparity between her feelings of insecurity and the perception others had of her as someone with a strong presence:

A harmless person then maybe yes I think that, she might perceive someone who is kind maybe a little shy and erm, ah but thus a little uninteresting to talk to….No I think that, (laughter), I think that uh, the picture that I'm describing, I'm not so sure that it's uh, actually correct. I… I don't know, maybe I have, I guess those are the feelings that I kind of have myself or think that people get the idea of this person but I've sure been refuted then many times anyway…eh,.

…but I think that I think I'm perceived as a person who doesn't take up much like space in the room or a person who, mmm, ah but is very modest and harmless is actually the image I see a person who is harmless eh… but also that, (laughter), eh, but maybe it's not, maybe rather that I think, maybe, the image that I think is not always, correct in how others see this person and maybe it will be, not a collision but, she goes in a way that she doesn't think is perceived, like goes briskly and maybe it does anyway, so there is some kind of uh, ah but some kind of uh, difference in how she feels that she is against how she might think others perceive her.

Toward the end of the second session, Klara experienced a shift in her self-perception, adopting a more positive outlook. However, this shift toward a more positive self-image contradicted the judgmental approach she previously held toward herself. Klara struggled to reconcile these opposing self-images: the familiar belief that she was flawed and the emerging understanding that she was, in fact, good enough:

Interviewer: How do you perceive this person in front of you right now?

Klara: eh, yes, but I would probably like to say that I look… ah, but I look quite relaxed and calm and, ah, but quite energetic too. I'm not this overworked mother of small children who just longs for the weekend, it doesn't feel right like. I'm still there eh, so, no, it still feels right, ah but she still looks quite lively, still eh, she looks like, ah but she still looks like she, she takes care of herself, takes care of her health. She has, uh, ah, she's not super pale despite the time of year. She comes out and ah, ah, she doesn't look like she has any major, problems like or it doesn't look like she has any problems either physically or mentally, she looks to have, she looks to have all quite good actually…

In contrast to Ann and Klara, Tim exhibited a less pronounced contrast between others' perceptions of him and his inner experiences. Rather than directly contrasting these aspects, Tim adopted a more detached perspective, observing himself from a distance. He focused on examining his overall life situation and how it had evolved over the years, as well as how he navigated various situations in his present life. This broader viewpoint allowed Tim to gain insights into his personal growth:

Interviewer: How do you perceive this person in front of you right now?

Tim: Well, but, I would probably say quite, relatively insightful, so, that like quite good at like doing, but priorities that are, but sustainable in, in everyday life both for the person but also people around, who can, prioritize away, things and prioritize things that, perhaps in the long term pay off more than in the short term, erm, and which perhaps are not all the way to the goal with it but which, nevertheless, have as a small, mission to, ah but make better long-term priorities.

### 3.4 Keeping emotions at bay

Participants expressed various strategies to manage their emotions. In the following excerpt, Ann cautiously approached challenging emotional experiences:

There is a… an everyday life as well which moves on rather eh, ah but quite fast and which then allows not to stop and sort of feel so much eh, inwardly… but… ah but it's clear there are unprocessed emotions eh, which is like ah but about love for others maybe eh, which she keeps at a distance also, sometimes feeling of loneliness which is not directly, eh, so it is not connected to real loneliness but there is still a like a feeling and a fear of loneliness that is quite strong eh… which she tries to keep at bay.

The virtual space could have potentially offered a secure setting for addressing challenging experiences. By adopting a third-person perspective and using the pronoun “she” instead of “I,” Ann may have found it more comfortable to delve into her vulnerabilities and engage in discussions about them. Similarly, Klara describes the development of an observational distance facilitated by the VR intervention as an out-of-body experience, allowing her to establish a distinct connection with herself that differs from traditional modes of self-perception:

Interviewer: What are you experiencing right now?

Klara: uh… well but it's… um, uh but it feels good I feel that I have, got uh I've gone into myself and, reflected on things like ah in a, not in a way that I never or yes, in a new way. I go inside myself, I often do anyway, but not in this way, when it gets like that, out of body in some way because it's like I've left my body and am looking at it from the outside, but I think, uh, I think it's a good feeling. I'm not sitting around feeling bad.

Overall, Klara acknowledged her emotions but tended to conceal them from others:

Interviewer: What is it then that you hold back?

Klara: It can be emotional states or, erm, ah if you're maybe a little, if you're a little half down for example or you've had a, ah you've had a fight with someone and you're sad about it, you can still… can you still put on a layer and act just like usual if you say, there's no reason in certain contexts for me to sit and, [sigh] ah what to say, be completely honest or open about how I feel, I think, without that can be, you can maybe, ah you can, you can have it inside.

Tim primarily directed his attention toward contextual factors that could account for his experiences, rather than fully immersing himself in the emotions themselves. When asked about his experiences and emotions, he tended to focus on general experiences in the past or future (there-then) and the factors that contributed to them, displaying minimal engagement with the present moment (here-and-now) experiences:

Interviewer: We now come back to here and now. What do you think the person in front of you is experiencing right now?

Tim: This person, I know, is experiencing a bit of stress, partly because it was an intense morning, but also because you stood up, on, on, involved in someone else's research when you actually had a lot of other things to do.

### 3.5 A judgmental approach to my own appearance

Ann and Klara had a tendency to critically evaluate their own appearance and experienced insecurity in relation to their physical appearance. In contrast, Tim had a more neutral stance toward his own appearance and tended to focus on aspects that reflected his social status.

Regarding first impressions, Klara expressed her anticipation of even more negative emotions when observing herself from an external perspective:

Klara: It wasn't as, I probably expected it to be harder, it wasn't as hard anyway…

Interviewer: In what way do you think it would have been more difficult?

Klara: [sigh] ah but one does have, one does have a built-in self-loathing (short laugh) in some way that one, uh… ah one is one's, one's, biggest critic, that one doesn't, like, there's always something that rubs something that one is not happy with and, uh, so, uh, one thinks about how others perceive you and… like that…… I wonder if I perceived, yes if I perceived[..] saw myself, if I had been someone else and met myself exactly as I do now… then, the question is what I would have liked about that, person.

Klara expressed being used to relating to herself in a critical manner and anticipated feelings of self-loathing when observing herself from an external perspective. However, she was somewhat surprised to find that it was not as difficult as she expected. Klara recognized that her tendency toward self-criticism could contribute to a skewed self-perception, while acknowledging that others might perceive her differently.

Ann experienced insecurity in relation to appearance and that even impacted her social relations:

Then there are other parts of me that are also connected to self-esteem and, looks that haven't been as erm, self-evident where I might have held back, er, and like that it also affected me a little bit in, ah but in relationship to other people that I don't think it's that super easy with, with contacts.

Ann felt exposed and uncomfortable when seeing herself in the VR-environment. Despite being observed only by herself, she felt exposed and experienced compulsions to conceal herself. This demonstrate the internalization of others' perspectives and their integration into the sense of self:

Interviewer: What do you think the person in front of you is experiencing right now?

Ann: (laughter), erm…… (laughter) she's feeling a bit uncomfortable at the moment erm… that's it.

Interviewer: Why is it uncomfortable?

Ann: erm, ah but maybe because it's very straight up then, that there's, erm, there's not much to hide behind, as I also think now that you've had two years of a pandemic and sat behind a screen and not uh seen much more than your face and then you see, now it's like standing straight up is an uncomfortable situation.

Regarding appearance, Tim oriented toward clothing and possessions that conveyed his social status or contextual information. He was attired in a manner that allowed him to blend into a particular context or situation, while simultaneously sensing a lack of complete authenticity or comfort:

Interviewer: How do you think the person in front of you perceives himself right now?

Tim: …as proper, it's probably one, it's probably still, not quite used to, this person to dress up in this way, although it might seem, in the context, quite like that, generic, that you don't seem to stand out in any particularly formal way. So this person is probably not at all used to having a shirt tucked into his trousers and, watch on his arm. That I think it's kind of a feeling that this isn't really,… weekday it is, but maybe it's not, a dress code or setting that he is used to.

### 3.6 Increased self-knowledge and acceptance with age

All the participants in the study were between their thirties and forties, and aging was perceived as having a positive influence on their self-knowledge and self-acceptance. Tim and Klara associated parenthood with a desire for personal growth and development:

It may well be connected with a lot, but partly because you, erm, get older, but also you getting children with whom you need to sort of talk, about serious and difficult things. Then you have to confront your own challenges and things that you yourself have found difficult, when explaining to them. (Tim)

Klara implied that her self-acceptance had improved with age, yet she continued to experience insecurities that she had anticipated that they would have diminished by this stage of life. This experience was accompanied by feelings of shame which become motivation for self-improvement. Klara expressed concerns about passing on these attitudes to her children, aspiring instead for them to develop a strong sense of self. As she perceived herself as a role model, particularly for her daughter, this influenced Klara's relating to oneself. Self-loathing became increasingly aversive, while self-acceptance became more desirable (appetitive):

You must have come to a, to a, time in life where you kind of more or less accept that it, [it] is the way I am, still, even though I might have thought that, when you're at this age then are you, one hundred percent at ease, but I still get exactly as I said before that, even now, you still have this fragility and, ah, will I be accepted by others eh, how do I look, how can I see, how can I, make myself better or something, eh. And there's a bit of shame in that I feel. I want to, as I have children myself and especially, one of them a girl, who I feel I don't want transfer that, that attitude to her, eh, but I want her to be, she should be confident in herself, ah, of course both children should be.

Tim experienced a sense of pride and significance when reflecting on his personal journey of self-development. He perceived himself as being in a favorable stage of life, which made it appealing for him to reflect on the progression of his life:

It's like a, also a, quite, merit, or meritorious is the wrong word but, to reflect on a, journey that one has sort of made anyway in one's, one's, emotional intelligence or one's own sort of feeling. It's quite, quite exciting to think about what, what you have felt and what you have, thought and how you have acted based on that, over the years, and you can do that as well when you can start thinking in decades instead of, years, so…

### 3.7 Acknowledging self-criticism fostered the development of self-compassion

The utilization of an external perspective within the VR-environment enabled participants to gain insight into their inclination toward self-criticism, thereby fostering the development of self-compassion. Klara was struck by the realization of how she tended to judge herself differently compared to how she approached others:

It strikes me a lot what I, how I judge myself in terms of appearance compared to what I do when I meet others.

Ann recognized and acknowledged the harshness with which she treated herself and realized that this self-critical attitude was not beneficial or helpful to her wellbeing:

But I do feel that it is a person who is a little hard on herself and that she could stop being that way, I think, eh, that's it.

Adopting an external perspective toward oneself could facilitate the recognition of being the target or victim of one's own harshness, possibly leading to an increase in aversive functions of that verbal behavior.

After removing the VR headset, Ann felt sense of longing for the self-connection experienced during the VR immersion:

I feel that I miss her a little… it's very rare that you see yourself that way.

This exemplified how the VR-intervention facilitated an experience of being connected with oneself (referred to as Me-Me-networking) and enabled a novel deictic relating.

## 4 Discussion

Through IPA, we aimed to explore participants' individual experiences regarding their sense of self (i.e., self-relating) during a perspective-taking intervention in VR. The intervention was designed to help participants approach previously avoided aspects of their deictic networks and, in doing so, facilitate hierarchical relating (i.e., observing their self-relating from a me-here-now perspective). It is important to note that the participants in this study were drawn from a non-clinical population and were not selected based on psychological problems. However, the observations from this study may still offer valuable insights with potential implications for clinical settings.

Seven group experiential themes were identified to describe participants' experiences about themselves; (1) The burden of thinking about how others perceive me, (2) Adjusting oneself according to social expectations vs. being true to oneself, (3) The mismatch between experiencing oneself from outside and inside, (4) Keeping emotions at bay, (5) The judgmental approach to my own appearance, and (6) Increased self-knowledge and acceptance with age (7) Acknowledging self-criticism fostered the development of self-compassion.

The participants extensively reflected on how they were perceived by others and recognized this as something they regularly did in their daily lives. This focus may have been partly influenced by the interview questions, which targeted a person's experience of themselves in relation to others (i.e., ME-OTHERS relating). However, during the VR intervention, participants also reflected on how their intense focus on others' perceptions affected them. It was experienced as a burden or a waist of energy, and it was associated with negative self-judgements and upward comparisons (i.e., experiencing oneself worse than others) but also a sense of meeting others expectations, or fitting in.

The participants' verbal expressions indicated that they became aware of how they were relating to themselves and others. Observing oneself from an external perspective (i.e., me-here-now observing me-there-now/then) may have facilitated a hierarchical position from which their own relating could be responded to, allowing for new derivation and the transformation of functions accordingly. First, the participants noticed how they related differently to themselves as compared to others, i.e., they noticed that they were being more harsh or critical toward themselves (Ann and Klara). When seeing oneself from the outside-perspective (i.e., responding to me-there-now from me-here-now), participants could relate to themselves both as a “victim' or a target of their own criticism and as a perpetrator who delivers the criticism. Relating to oneself as a victim evoked self-compassion and led to defending oneself against self-loathing. “It's a person who is a bit hard on herself, and I think she could stop being that way”. Thus, observing oneself from the outside while acknowledging malicious self-talk may have helped to expose its harmful effects.

Another instance of comparative relating that was perceived as helpful was between the old narrative and the visual representation seen in the VR. Two participants (Ann and Klara) noticed that their self-stories were inconsistent with how they perceived themselves through the VR-headset (e.g., feeling like an unconfident person vs. seeing a calm and confident person). Furthermore, one participant (Ann) found that feedback she had received from others that differed from her own perception was confirmed through the VR-film (e.g., “I have been told that I have a strong presence, when I speak in front of people.”). This also made her see how resistant she was to external positive feedback. The perceived incoherence may have forced her to incorporate these ideas in a more complex story e.g., “I think that maybe the image that I have [about myself] doesn't always correspond to how others see this person [me]”. Becoming aware of one's resistance to positive feedback could be a clinically significant insight, considering that addressing negative self-perceptions is often challenging due to their strong resistance to change or external validation (Beck Aaron et al., 1979; Swann et al., 1987; Young et al., 2003).

Some prompts encouraged the participants to shift temporal perspective which promoted reflection of development over time. In general, aging was associated with increased self-knowledge and self-acceptance, which is in line with longitudinal evidence suggesting that self-esteem tends to increase from adolescence to middle adulthood, and then decrease into old age (Orth and Robins, 2014). Participants described teenage years as a transition period when awareness of others' perspective developed and was more prominent. “Fitting in” became important, and it had remained to be so. From the participants' current adult perspective, this was experienced as a conflict; the ideal was an adult who knows and accept oneself, yet the importance of fitting in and gaining other's acceptance was still an inseparable aspect of self. All participants felt that they adjusted themselves to fit in and for some participants (Ann and Klara) this was at odds with their sense of authenticity.

Participants differed in how they conceptualized the way their self relates to and is shaped by the environment. Some narratives included a “true self” (Klara) that could be more or less expressed in different situations, e.g., “I don't know if I can say that I would always be hundred percent myself.” Whereas one participant (Tim) attributed his sense of self strongly to the context. He expressed little fixed ideas about himself, and focused on different factors that impacted on his experiences. He acknowledged that his own-, and others perception of him varied with the context. ”*…life goes up and down and now it is, is it an opportunity there, a context where it is quite easy, where there are, things that are fun around you and there is, time and space and even if things, can be stressful in everyday life, so it is not beyond control and then it can be easy to find contact with what you feel, but if it had been a different situation where you may have had less control or where it has, as it were, been on people other than yourself what, how the outcome of something is then maybe it would have been different…”*

Overall, the intervention may have facilitated verbal tracking of the sources of control over behavior (i.e., monitoring how behavior, including thoughts and feelings, is influenced by internal and external contextual cues). An example of this was Klara scrutinizing how she modified her facial expressions in the 3D film to appear more self-confident: “…*here she knows she's being filmed, uh. I thought it was very interesting to see the very first seconds… she stands, no, she doesn't make an effort, she's more relaxed in the face, eh. Then she tenses, she pulls the lower jaw forward because she knows she has an overbite … so she has a different look than she had at the beginning, eh, as she thinks she, thinks she looks, that she simply looks better, that she should show, as it were, that she is, I am, I have self-confidence”*. Successful tracking of the sources of control over behavior has been described as foundational in establishing a healthy sense of self (Barnes-Holmes et al., 2018). This phenomenon has also been referred to as context sensitivity, which has been proposed as an important goal in therapeutic work (Villatte et al., 2015). For instance, this could involve a person becoming aware of their tendency to have self-critical thoughts in certain situations, rather than remaining stuck with the thought, “I am not good enough”. Increasing awareness of one's responses to different contextual cues also includes the ability to adopt a hierarchical perspective from which the sources of control can be observed. In future studies, it might be interesting to explore verbal tracking within participants' self-narratives in more detail, particularly in clinical populations.

In summary, assessing self-relating while viewing oneself from an outside perspective in the VR environment provided interesting and potentially clinically valuable insights into participants' deictic relating. Participants were able to respond from a distinct position to the coordination between “me” and self-critical labels. This, for instance, led to questioning their tendency to be harsh toward themselves. Moreover, participants recognized their tendencies to adopt others' perspectives on themselves and to adjust their behavior based on assumed expectations of others. Viewing themselves from the outside also enabled them to detect the double standards they applied when relating to themselves vs. others (e.g., judging their own appearance in a way they would never judge anyone else). The intervention further highlighted inconsistencies between how participants perceived themselves from the outside and from within. For instance, they responded less negatively to themselves in the VR environment. This highlights a potential therapeutic advantage of the VR intervention: its ability to create a safe space for participants to explore their self-narratives from the distance (e.g., reflecting “her” instead of “me”) and alternative self-narratives without the emotional intensity or resistance that can arise in real-life or traditional settings. This finding aligns with research suggesting that distanced self-reflection can reduce emotional reactivity and facilitate cognitive restructuring (Kross and Ayduk, 2011).

Also previous research have demonstrated VR's potential to facilitate novel self-dialogues. For instance, in one study participants first embodied an adult figure providing comfort to a virtual child and then switched perspectives to embody the child, experiencing compassion from the adult's viewpoint. This approach significantly increased self-compassion compared to a control group that observed the same interaction from a third-person perspective (Falconer et al., 2014). Similarly, Osimo et al. (2015) explored a VR paradigm where participants alternated between embodying themselves and Sigmund Freud to discuss personal problems. This setup allowed participants to provide themselves with advice from Freud's perspective. The study found that participants experienced improved mood when embodying Freud compared to embodying themselves (Osimo et al., 2015). In contrast to these previously utilized VR methods, the current VR intervention used a technically simple solution to provide an external perspective of oneself. This method did not rely on avatars or a sense of embodiment (i.e., substituting the participant's body with a virtual body), making it highly accessible and straightforward to implement in clinical practice.

The VR intervention shares some overlap with established self-reflection methods, such as video feedback and mirror exposure. However, participants appeared to respond differently to seeing 3D representations of themselves compared to viewing themselves in a mirror or on traditional 2D video. Similar to mirror gazing (Lipson et al., 1983), participants initially focused on their appearance at the start of the intervention. Importantly, they did not only evaluate their appearance but also reflected on their relationship to their appearance—how their judgmental attitudes toward their physical features impacted them. For instance, one participant noted how insecurity about her appearance affected her social relationships. As the intervention progressed, participants shifted their focus to other aspects of themselves, developing a sense of connection with their self-representation. This is exemplified by one participant's remark immediately after removing the headset: “I feel that I miss her a little… it's very rare that you see yourself that way.” Meeting oneself in the VR environment generally evoked feelings of self-compassion, contrasting with the self-criticism often associated with viewing photographs of oneself (Diefenbach and Christoforakos, 2017). Encouraging participants to notice and reflect on their relationship with their physical features (i.e., hierarchical relating, such as: “I notice how I relate to my physical features and how it affects me”) may be key in mitigating undesirable effects of interventions that involve observing oneself from an external perspective in VR. Although this study did not provide statistical data to support firm conclusions, none of the participants reported adverse effects or engaged in sense-making that revealed plausible paths to such outcomes.

The current findings suggest potentially beneficial clinical implications of the VR intervention. It could serve as a valuable tool for clinicians in assessing and influencing deictic relating. The fact that self-related processes are addressed in most established psychological treatment approaches underscores the centrality of deictic relating in influencing psychological wellbeing. For example, a key aim of Cognitive Therapy is to challenge and modify negatively biased self-evaluations (Beck Aaron et al., 1979). Similarly, Schema Therapy provides a perspective on self-relating through maladaptive schemas about the self, such as feelings of inadequacy or unworthiness (Young et al., 2003). Techniques like imagery rescripting in schema therapy help individuals challenge and reframe these core beliefs, which are often rooted in early attachment experiences. Similar to the intervention used in the current study, Acceptance and Commitment Therapy promotes hierarchical relating to self-related thoughts and emotions, reducing rigid control and avoidance strategies (Hayes et al., 2011) Compassion-Focused Therapy targets self-criticism and shame through imagery and cognitive restructuring, emphasizing the development of self-compassion (Gilbert, 2009). While each approach offers its own conceptualization and methods for addressing self-criticism, shame, and negative self-narratives, they also overlap significantly. Central to many of these approaches is increasing awareness of negative self-relating and enabling individuals to change how they relate to their self-narratives, thereby fostering new and more adaptive ways of self-relating. The VR intervention appears to offer promising possibilities for influencing these processes.

However, it is important to acknowledge that the current insights were drawn from a small, non-clinical sample with a narrow age range and no quantitative data. As such, they are exploratory in nature and not intended to be generalized, and not sufficient to draw definitive conclusion about the effectiveness of the intervention, but rather to inform the future development of VR interventions targeting deictic relating. Future research should utilize idiographic methods to examine the processes involved and effects of the VR intervention on individuals facing psychological challenges related to their sense of self, including conditions like depression or eating disorders.

## Data Availability

The ethical application was formulated in such a way that publishing the datasets would contradict what was ultimately approved. For example, the informed consent states, “All information that comes to us will be processed in such a way that no unauthorized persons get access to it.” Additionally, sharing transcriptions of the interview data could risk violating participants' privacy. Requests to access the datasets should be directed to essi.sairanen@kau.se.
